# Impact of Race on Outcomes of Advanced Stage Non-Small Cell Lung Cancer Patients Receiving Immunotherapy

**DOI:** 10.3390/curroncol30040321

**Published:** 2023-04-18

**Authors:** Melisa Pasli, Radhamani Kannaiyan, Praveen Namireddy, Paul Walker, Mahvish Muzaffar

**Affiliations:** 1 Brody School of Medicine at East Carolina University, Greenville, NC 27834, USA; 2 Division of Hospital Medicine, Eat Carolina University Health, 2100 Stantonsburg Road, Greenville, NC 27834, USA; 3 Division of Hematology/Oncology, East Carolina University, Greenville, NC 27834, USA; 4 Circulogene, Birmingham, AL 35209, USA

**Keywords:** non small cell lung cancer, immunotherapy, race

## Abstract

Background: The impact of race in advanced stage non-small cell lung cancer (NSCLC) patients treated with immune checkpoint inhibitors (ICIs) is conflicting. Our study sought to examine racial disparities in time to treatment initiation (TTI), overall survival (OS), and progression-free survival (PFS) using a population that was almost equally black and white. Methods: This was a retrospective cohort study of stage IV NSCLC patients > 18 years receiving immunotherapy at our center between 2014 and 2021. Kaplan—Meier curves and the multivariate Cox proportional hazards model determined the predictors of OS and PFS. Analyses were undertaken using IBM PSAW (SPSS v.28). Results: Out of 194 patients who met the inclusion criteria, 42.3% were black (*n* = 82). In the multivariate analysis, there was no difference in PFS (HR: 0.96; 95% CI: 0.66,1.40; *p* = 0.846) or OS (HR: 0.99; 95% CI: 0.66, 1.48; *p* = 0.966). No difference in treatment selection was observed between white and black patients (*p* = 0.363), nor was there a difference observed in median time to overall treatment initiation (*p* = 0.201). Conclusions: No difference was observed in OS and PFS in black and white patients. Black patients’ reception of timelier immunotherapy was an unanticipated finding. Future studies are necessary to better understand how race impacts patient outcomes.

## 1. Introduction

Lung cancer is the leading cause of cancer deaths, and is responsible for approximately 25% of cancer-related deaths in both women and men [1]. The most common type of lung cancer is non-small cell lung cancer (NSCLC), accounting for approximately 85% of all lung cancers [2]. Approximately 55% of patients [3] are diagnosed at an advanced stage due to lack of clinical symptoms [2]. Over the last few decades, the systemic therapy options have evolved for stage IV lung cancer from platinum-based chemotherapy to a more targeted, personalized approach [3,4]. Immune checkpoint inhibitors (ICI) and targeted therapy, alone or in combination with other agents, have revolutionized cancer therapy [5,6] and greatly enhanced overall survival (OS) rates [7]. The combination of chemotherapy with ICIs acts synergistically, and is deemed first-line therapy for a majority of patients with advanced stage NSCLC, irrespective of PD-L1 expression [7].

Most of these advances are based on clinical trials underrepresenting ethnic minorities [8,9]. Black patients demonstrate a higher rate of stage IV cancer [10] and have a decreased 5-year survival rate [10,11] when compared to white patients. Black patients have a higher propensity to develop lung cancer at a younger age and with fewer pack-years of smoking [12,13]. Research has also highlighted biologic differences in lung cancer among black and white patients [14]. Black patients with lung cancer exhibit a lower tumor mutation burden (TMB) [15] and an inflammatory protein profile [16] compared to white patients, suggesting potential differences in ICI response [15]. This difference in ICI response may in part be due to PD-L1’s polymorphism in different races or the habits of different people.

There are known racial disparities in treatment reception in most cancers, and lung cancer is no exception [17,18]. Various factors contribute to barriers in care, such as the vicinity of treatment facilities [19], lack of transportation opportunities [19], lack of medical insurance [10], and medical mistrust [19]. These factors have been extensively reported in minority patients in comparison with their nonminority counterparts [20].

Previously conducted studies have demonstrated a positive impact of equal access anticancer treatment on racial disparities in NSCLC patients, but these studies had a lower percentage of black patients in the overall cohort [21,22]. Our study cohort has a larger proportion of black patients [2,23,24], allowing us to study the impact of race on immunotherapy-based treatment regimens for advanced lung cancer in an equal access health care setting. To our knowledge, this study presents the greatest ratio of black patients, and is the first study focusing on racial disparities in advanced stage NSCLC patients regarding time-to-treatment initiation (TTI). Our hypothesis was that no racial differences would be evident regarding TTI, OS, or PFS between black and white patients.

## 2. Methods

This was an IRB-approved retrospective study of stage IV NSCLC patients aged >18 years who received immunotherapy-based regimens consisting of anti–PD-1/PD-L1 agents at ECU Health between 2014 and 2021. Follow-up data were recorded until 9 March 2022. A total of 317 patients were screened and 194 patients were deemed eligible. Inclusion criteria included those patients aged >18 years with a diagnosis of NSCLC. Our exclusion criteria consisted of those with small cell lung cancer (SCLC), the presence of other concurrent cancers, and those who solely received chemotherapy regimens. As this was a retrospective study, sample size determination was based on the inclusion of all eligible cases. Only records in existence at the time of the IRB review and approval were accessed for review. Medical records were reviewed by study staff at East Carolina University and ECU Health.

Demographics and baseline characteristics were analyzed utilizing descriptive statistics. Demographic characteristics collected included sex, age at diagnosis, race, stage at diagnosis, insurance type, Eastern Cooperative Oncology Group (ECOG) status at immunotherapy initiation, histological subtype of cancer, marital status, Charlson comorbidity index (CCI), median number of pack-years, cumulative presence of brain metastasis, platinum-based combination ICI therapy reception, hospice enrollment, metformin use, and statin use at time of diagnosis. CCI was utilized to determine the underlying comorbidities of our patient cohort (cancer-free CCI) [25].

Analyses were conducted utilizing IBM PASW (SPSS Version 28) (Armonk, NY, USA: IBM Corp.). Chi-square tests were used to analyze racial differences in categorical variables and to compare the median time from date of diagnosis to immunotherapy reception and overall cancer treatment reception. The primary endpoints measured included overall survival (OS) and progression-free survival (PFS). OS and PFS were calculated with Kaplan—Meier analysis. OS was calculated as the period from the day of immunotherapy treatment initiation until the day of death. PFS was defined as the time of immunotherapy initiation to radiological progression or death, whichever was first. A multivariate Cox proportional hazards model was applied to determine predictors of PFS and OS. Hazard ratios and 95% confidence intervals were reported with *p*-value < 0.05 indicating significance. Only black and white patients were included in the analysis. Any patients with missing variables in the multivariate regression were excluded from the analysis. Therefore, this explains the sample size discrepancy between number of patients in Table 1, Table 2, Table 3 and Table 4. PD-L1 status was not assessed in the multivariate analysis due to missing values that may have impacted the results.

Our patient cohort, consisting of white and black stage IV NSCLC patients who received either first-line or subsequent-line immunotherapy, was divided into two groups: TTI > 20 days and TTI ≤ 20 days, a similar methodology as that described by Azzouqa et al. [26]. Kaplan—Meier log-rank tests were computed to analyze OS between the groups. A Cox regression multivariate model was computed and included age at diagnosis, sex, race, insurance type, marital status, CCI, pack-years, and histological subtype of cancer.

## 3. Results

A total of 317 patients were analyzed and screened. 194 were deemed eligible for this study. As described in Table 1, our cohort consisted of 54.1% white patients (*n* = 105), 42.3% black patients (*n* = 82), 0.5% Asian patients (*n* = 1), 2.6% other ethnicity (*n* = 5), and 0.5% unknown (*n* = 1). Our cohort had 76.8% adenocarcinoma (*n* = 149), 17.5% squamous cell carcinoma (*n* = 34), and 5.7% other (including not otherwise specified (NOS), adenosquamous, sarcomatoid, and poorly differentiated) (*n* = 11) patients. The median age of diagnosis was 64.48. A total of 20.2% of our cohort had Medicaid insurance, 63.4% experienced irAEs (immune-related adverse events), and 66.5% received platinum-based combination immunotherapy. At the censoring date of the study, 25/105 (23.8%) white patients and 20/82 (24.4%) black patients were still alive. Most of our patients (91.8%) had a smoking history. A total of 17.5% of patients received immunotherapy as second-line treatment. Pembrolizumab (74.7%), nivolumab (13.4%), nivolumab plus ipilimumab (1.5%), atezolizumab (8.8%), and durvalumab (1.5%) were the ICIs most commonly administered.

**Table 1:** Demographic Characteristics of Stage IV NSCLC Patients (*n* = 194).

Characteristics	Frequency (*n*)	%
Sex		
Female	87	44.8
Male	107	55.2
Age at diagnosis ^a^	64.5 (35.4–92.6)	
Race		
White	105	54.1
Black	82	42.3
Asian	1	0.5
Other/Unknown	6	3.1
Insurance type		
Medicare	117	60.6
Medicaid	39	20.2
Private	31	16.1
VA	3	1.6
None	3	1.6
Missing	1	
ECOG at ICI initiation		
0	23	14.9
1	78	50.6
2	46	29.9
3	7	4.5
Missing	40	
Histological type		
Adenocarcinoma	149	76.8
Squamous cell carcinoma	34	17.5
Other	11	5.7
Marital Status		
Married	89	46.1
Widowed	34	17.6
Single	33	17.1
Divorced/Legally Separated	37	19.2
Missing	1	
CCI		
0	9	4.6
1	28	14.4
2	47	24.2
≥3	110	56.7
Smoker at diagnosis		
Yes	77	39.7
No	117	60.3
Former smoker		
Yes	178	91.8
No	16	8.2
Pack-years	30 (0–160)	
0–30	94	50.5
31–60	64	34.4
>60	28	15.1
Missing	8	
Hospice enrollment		
Yes	98	64.5
No	54	35.5
Not applicable	42	
If enrolled, hospice enrollment for at least a week prior to day of death		
Yes	54	55.1
No	44	44.9
Unknown	3	
Experienced irAE		
Yes	123	63.4
No	71	36.6
Cumulative brain metastasis		
Yes	95	49.0
No	99	51.0
ICI selection		
Pembrolizumab	145	74.7
Nivolumab	26	13.4
Nivolumab plus Ipilimumab	3	1.5
Atezolizumab	17	8.8
Durvalumab	3	1.5
Platinum-based combination ICI therapy		
Yes	129	66.5
No	65	33.5
PD-L1 status		
<1%	66	44.3
1–49%	29	19.5
≥50%	54	36.2
Missing	45	
Metformin use		
Y	15	7.7
N	179	92.3
Statin use		
Y	90	46.4
N	104	53.6

^a^ Date ranges are reported in median (range). Abbreviations: ECOG, Eastern Cooperative Oncology Group; ICI, immune-checkpoint inhibitor; CCI, Charlson comorbidity index; irAE, immune-related adverse event; PDL-1, Programmed Cell Death Ligand 1.

In the chi-square analysis (Table 2), significant associations were found between race and marital status (*p* < 0.001), CCI (*p* = 0.030), pack-years (0–30, 31–60, >60) (*p* = 0.023), age at diagnosis (<65, 65–74, >75) (*p* = 0.036), and insurance type (*p* = 0.047). There was no significant difference in experiencing irAEs (*p* = 0.802), platinum-based combination ICI therapy reception (*p* = 0.239), presence of cumulative brain metastasis (*p* = 0.361), treatment selection (*p* = 0.363), PD-L1 status (*p* = 0.222), hospice enrollment (*p* = 0.279), ECOG status at immunotherapy initiation (*p* = 0.123), being a smoker at diagnosis (*p* = 0.349), metformin use (*p* = 0.819), and statin use (*p* = 0.220) between white and black patients. Black patients had significantly fewer median pack-years (*p* = 0.025) than white patients.

**Table 2:** Qualitative and Quantitative Characteristics by Race in Stage IV NSCLC patients (*n* = 187) ^a,b^.

*n*, %			
Characteristics	Black (*n* = 82)	White (*n* = 105)	*p*-Value ^c^
Sex			
Female	37 (45.1)	45 (42.9)	0.757
Male	45 (54.9)	60 (57.1)	
Age at diagnosis			
<65	45 (54.9)	51 (48.6)	0.036
65–74	28 (34.1)	27 (25.7)	
≥75	9 (11.0)	27 (25.7)	
Age at diagnosis	63.9 (35.4–89.7)	65.6 (45.0–92.6)	0.600
Insurance type			
Medicare	48 (58.5)	64 (61.5)	0.047
Medicaid	23 (28.0)	15 (14.4)	
Private	9 (11.0)	21 (20.2)	
VA	0 (0.0)	3 (2.9)	
None	2 (2.4)	1 (1.0)	
Missing = 1			
ECOG at ICI initiation			
0	11 (16.9)	11 (13.3)	0.123
1	28 (43.1)	45 (54.2)	
2	25 (38.5)	21 (25.3)	
3	1 (1.5)	6 (7.2)	
Missing = 39			
Histological type			
Adenocarcinoma	63 (76.8)	81 (77.1)	0.994
Squamous cell carcinoma	14 (17.1)	18 (17.1)	
Other	5 (6.1)	6 (5.7)	
Marital Status			
Married	26 (31.7)	60 (57.1)	<0.001
Widowed	17 (20.7)	16 (15.2)	
Single	25 (30.5)	7 (6.7)	
Divorced/Legally separated	14 (17.1)	22 (21.0)	
CCI			
0	5 (6.1)	4 (3.8)	0.030
1	14 (17.1)	13 (12.4)	
2	25 (30.5)	17 (16.2)	
≥3	38 (46.3)	71 (67.6)	
Smoker at diagnosis			
Yes	36 (43.9)	39 (37.1)	0.349
No	46 (56.1)	66 (62.9)	
Former smoker			
Yes	74 (90.2)	98 (93.3)	0.440
No	8 (9.8)	7(6.7)	
Pack-years range			
0-30	48 (60.0)	40 (40.4)	0.023
31–60	24 (30.0)	39 (39.4)	
>60	8 (10.0)	20 (20.2)	
Pack-years	25/(0–100)	40/(0–160)	0.025
Missing = 8			
Hospice enrollment			
Yes	39 (60.9)	57 (69.5)	0.279
No	25 (39.1)	25 (30.5)	
Missing or not applicable = 41			
Experienced irAE			
Yes	53 (64.6)	66 (62.9)	0.802
No	29 (35.4)	39 (37.1)	
Cumulative brain metastasis			
Yes	43 (52.4)	48 (45.7)	0.361
No	39 (47.6)	57 (54.3)	
Platinum-based combination ICI therapy			
Yes	59 (72.0)	67 (63.8)	0.239
No	23 (28.0)	38 (36.2)	
ICI Selection ^d^			
Pembrolizumab	65 (79.3)	76 (72.4)	0.363
Nivolumab	8 (9.8)	17 (16.2)	
Nivolumab plus Ipilimumab	1 (1.2)	1 (1.0)	
Atezolizumab	8 (9.8)	8 (7.6)	
Durvalumab	0 (0.0)	3 (2.9)	
PDL-1 status			
<1%	31 (47.7)	33 (42.3)	0.222
1–49%	15 (23.1)	12 (15.4)	
≥50%	19 (29.2)	33 (42.3)	
Missing = 44			
Metformin use at diagnosis			
Y	7 (8.5)	8 (7.6)	0.819
N	75 (91.5)	97 (92.4)	
Statin use at diagnosis			
Y	34 (41.5)	53 (50.5)	0.220
N	48 (58.5)	52 (49.5)	
Time to ICI initiation (days)	27 (2–318)	37 (0–278)	0.030
Time to overall treatment initiation (days)	22 (1–141)	27 (0–183)	0.201

^a^ Non-black, non-white patients and missing data were removed from analysis due to small percentage composition (3.6%). ^b^ All numerical data and date ranges are reported in median (range). ^c^ Chi-square tests were utilized to compare both categorical variables and median values. ^d^ If more than one line of ICI was given, the first immunotherapy was listed. Abbreviations: ECOG, Eastern Cooperative Oncology Group; ICI, immune-checkpoint inhibitor; CCI, Charlson comorbidity index; irAE, immune-related adverse event; PDL-1, Programmed Cell Death Ligand 1.

**Figure 1:**
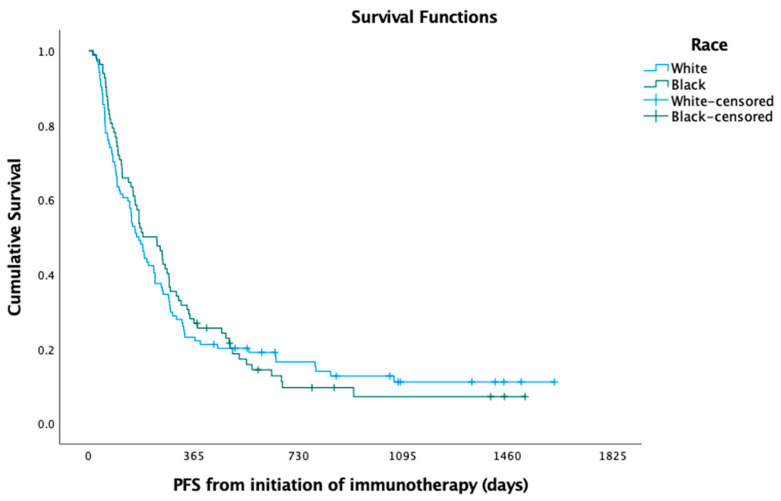
Progression-free survival (PFS) among black versus white patients.

**Figure 2:**
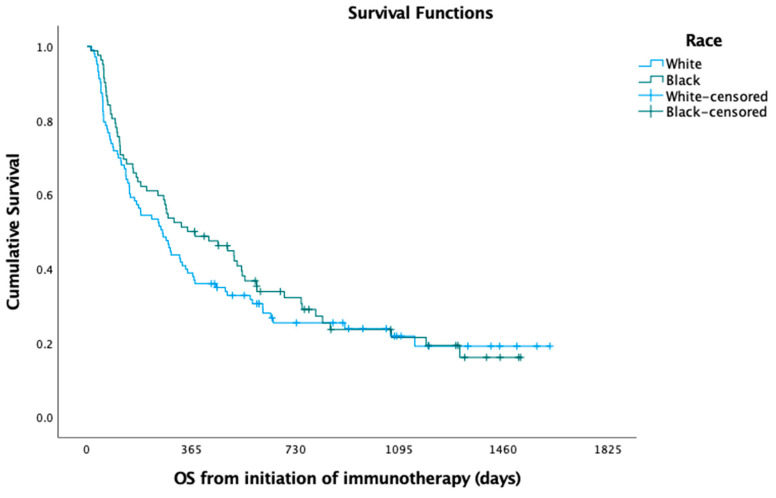
Overall survival (OS) among black versus white patients.

In the Kaplan—Meier analysis, no difference was observed between white and black patients with respect to median PFS (5.49 versus 6.22 months; log-rank *p* = 0.725) (Figure 1) and OS (8.72 versus 11.61 months; log-rank *p* = 0.397) (Figure 2). In the Cox regression multivariate analysis, no difference in PFS (HR = 0.96; 95% CI: 0.66, 1.40; *p* = 0.846) (Table 3) or OS (HR = 0.99; 95% CI: 0.66, 1.48; *p* = 0.966) (Table 4) was evident when adjusting for sex, age at diagnosis, marital status, histology, pack-years, insurance, and Charlson comorbidity index (CCI). In the Kaplan—Meier analysis, median OS did not differ between white and black patients, respectively, when stratifying the patient cohort by PD-L1 status to assess racial disparities in these subgroups (PD-L1 < 1%; log-rank *p* = 0.181; 7.47 months versus 10.03 months; PD-L1 1–49%; log-rank *p* = 0.361; 6.18 months versus 16.97 months; PD-L1 > 50%; log-rank *p* = 0.587; 10.79 months versus 8.98 months). The median time to overall cancer treatment (27 days versus 22 days; *p* = 0.201) did not differ, but there was a significant difference in time to immunotherapy initiation (37 days versus 27 days; *p* = 0.030) between white and black patients, respectively.

**Table 3:** Multivariate Association between PFS and Demographic Characteristics of Stage IV NSCLC Patients.

Covariate	Level	Number	Multivariate Model	
			HR (95% CI)	HR *p*-Value
Race	Black	78	0.96 (0.66, 1.40)	0.846
	White	93	Reference	Reference
Type of Cancer	Squamous	28	2.08 (1.34, 3.23)	0.001
	Non-squamous	143	Reference	Reference
Sex	Male	97	Reference	Reference
	Female	74	0.81 (0.57, 1.15)	0.238
CCI	0,1	31	Reference	Reference
	2	39	0.74 (0.41, 1.33)	0.308
	≥3	101	0.75 (0.40, 1.39)	0.355
Insurance	Government	143	Reference	Reference
	Private	28	1.03 (0.61, 1.75)	0.906
Marital Status	Married	77	Reference	Reference
	Single	30	0.91 (0.53, 1.57)	0.731
	Widowed	32	0.66 (0.40, 1.07)	0.091
	Divorced/Legally separated	32	1.56 (0.97, 2.52)	0.068
Age at diagnosis		171	1.03 (1.01, 1.06)	0.011
Pack-years		171	1.00 (0.99, 1.00)	0.339

Abbreviations: CCI, Charlson comorbidity index.

**Table 4:** Multivariate Association between OS and Demographic Characteristics of Stage IV NSCLC patients.

Covariate	Level	Number	Multivariate Model	
			HR (95% CI)	HR *p*-Value
Race	Black	78	0.99 (0.66, 1.48)	0.966
	White	92	Reference	Reference
Type of Cancer	Squamous	28	1.68 (1.06, 2.66)	0.026
	Non-squamous	142	Reference	Reference
Sex	Male	97	Reference	Reference
	Female	73	0.89 (0.61, 1.31)	0.566
CCI	0,1	31	Reference	Reference
	2	39	0.75 (0.39, 1.45)	0.394
	≥3	100	0.93 (0.49, 1.79)	0.835
Insurance	Government	142	Reference	Reference
	Private	28	1.27 (0.74, 2.17)	0.384
Marital Status	Married	77	Reference	Reference
	Single	30	0.70 (0.38, 1.31)	0.269
	Widowed	31	0.78 (0.46, 1.32)	0.349
	Divorced/Legally separated	32	1.85 (1.14, 3.02)	0.013
Age at diagnosis		170	1.02 (0.99, 1.05)	0.138
Pack-years		170	1.00 (0.99, 1.00)	0.529

Abbreviations: CCI, Charlson comorbidity index.

In the multivariate analysis, patients with squamous cell carcinoma had more than twice the risk of disease progression than those with nonsquamous histology (HR = 2.08; 95% CI: 1.34, 3.23; *p* = 0.001). PFS was also significantly associated with age at diagnosis (HR = 1.03; 95% CI: 1.01, 1.06; *p* = 0.011). There were no statistically significant associations between PFS and race, sex, CCI, insurance type, marital status, or pack-years. In the multivariate analysis, OS was significantly associated with cancer histological subtype (non-squamous versus squamous histology) (HR = 1.68; 95% CI: 1.06, 2.66; *p* = 0.026) and marital status (higher risk of death in divorced compared to married (HR = 1.85; 95% CI: 1.14, 3.02); *p* = 0.013). The risk of death was 1.85 times greater in divorced patients than in married patients. There were no significant associations between OS and race, sex, CCI, insurance type, age at diagnosis, or pack-years.

The median follow-up time for our cohort was 11.5 months. There was no significant difference in 12-month OS rates between black (50.0%) and white (38.8%) patients (*p* = 0.128). This also held true when evaluating 24-month OS rates between black (24.4%) and white (18.4%) patients (*p* = 0.325). The 12-month PFS rates were similar among black (28.0%) versus white (23.1%) patients (*p* = 0.439). Additionally, there was no significant difference in 24-month PFS rates among black (7.3%) and white (12.5%) patients (*p* = 0.247).

## 4. Discussion

Our study sought to examine the impact of race on the outcomes of patients with advanced lung cancer treated with an immunotherapy-based regimen. To our knowledge, this study is one of few with almost equal representation of black and white patients. Few studies provide significant representation of black patients, and these studies lack detail on parameters impacting access to care, including TTI.

Although a study assessing the relationship between race and all-cause mortality between white and black NSCLC patients receiving ICIs has been conducted, the patient cohort was only composed of 12.6% black patients [2]. In addition, a study by Deng et al. focusing on racial disparities in time to immunotherapy reception for stage IV NSCLC patients was composed of 87.7% white patients [24]. A study examining PFS and OS in stage IV NSCLC lung cancer patients receiving first-line pembrolizumab identified no difference in PFS or OS between white and black patients. However, this study was limited by its smaller sample size (*n* = 136) and lower percentage of black patients. [23]. Our study distinguishes itself in that 42.3% of our cohort was composed of black patients, exceeding the existing literature in terms of percentage of black patients in the overall cohort [22,23].

Our study showed no difference in median PFS (6.22 months versus 5.49 months; log-rank *p* = 0.725) and OS (11.61 months versus 8.72 months; log-rank *p* = 0.397) between black and white patients, respectively. These findings are similar to previously conducted studies [22,23] on advanced stage NSCLC patients receiving immune checkpoint inhibitors (ICIs). The lack of significance noted between black and white advanced NSCLC patients for 12-month and 24-month PFS and OS rates is consistent with a study conducted by Nazha et al. [22].

Clinical outcomes of white versus black patients with advanced NSCLC receiving immunotherapy have previously been investigated, but had a patient cohort composed of only 29.5% black patients [22]. Another important distinction is that 66.5% of our cohort received pembrolizumab in combination with platinum-based chemotherapy (or platinum-based chemotherapy in combination with ICB), which is now first-line treatment for advanced NSCLC patients [7,27]. Thus, our study, in addition to having a more representative black population, also represents a regimen which is now the standard of care. Our study additionally distinguishes itself in that patients who received immunotherapies other than pembrolizumab were also included [23].

Consistent with other studies [22], no significant differences in PD-L1 expression were found between white and black patients (*p* = 0.222, chi-square). High PD-L1 expression has been shown to be associated with improved outcomes for patients treated with ICIs [22,28]. However, to our knowledge, there are not many studies comparing OS in white and black patients based on PD-L1 status to assess racial disparities in these subgroups. In our study, median OS in the Kaplan—Meier analysis did not differ between white and black patients when stratifying the patient cohort by PD-L1 status. As such, PD-L1 status did not seem to impact the similar survival between white and black patients.

Azzouqa et al. examined TTI in stage I-IV NSCLC patients, and demonstrated that TTI ≤ 20 days resulted in worse OS than in stage IV patients with a TTI >20 days. Although it yielded meaningful results, this study did not include race as a factor [26]. We added to these results by incorporating race into a separate multivariate analysis and controlling for additional variables. Patients with TTI >20 cohort had a lower risk of death than TTI ≤ 20 cohort (HR = 0.671; 95% CI: 0.47,0.97; *p* = 0.031). In the Kaplan—Meier analysis, median OS increased in patients with TTI > 20 days (12.47 months versus 8.52 months; log-rank *p* = 0.013) for overall cancer treatment when compared to the TTI ≤ 20 cohort. TTI for first immunotherapy reception demonstrated no difference in median OS in the TTI > 20 cohort (8.98 months versus 10.99 months; log-rank *p* = 0.776) when compared to the TTI ≤ 20 cohort. As such, this did not alter established findings.

For advanced stage IV NSCLC patients, relatively delayed immunotherapy or chemotherapy treatment initiation was found to be paradoxically associated with lower risk of death [29]. This has been partially explained by the “sicker quicker phenomenon”, where more aggressive tumors result in patients becoming more symptomatic and thus receiving earlier treatment, but the aggressive nature of the advanced cancer still results in overall worse prognosis. This study, however, did not have data on smoking information, comorbidities, and did not assess racial differences [29]. Another study reported TTI < 35 days was associated with improved survival in metastatic NSCLC patients who survived more than a year, whereas this was not the case for patients who survived less than a year. Patients with more manageable metastatic disease derived a significantly pronounced benefit from accelerated treatment initiation, whereas those patients with a more lethal malignancy profile did not [30].

Studies have shown an association between increased mortality and delay in treatment reception, but this was observed in patients with non-metastatic lung cancer [31,32]. This may indicate that delay in treatment initiation in early-stage disease enables the progression of curable disease into an incurable disease [33]. The ideal time for treatment remains controversial. Future studies are needed to better understand how the timing of treatment initiation impacts survival and overall prognosis in different cancer subtypes and stages, as the results are not conclusive [32,34].

Zyczynski et al. demonstrated similar TTI between black and white patients for advanced NSCLC [34]. Similarly, in our study, median time to overall treatment initiation did not differ by race. Conversely, our study demonstrated that median time from diagnosis to immunotherapy treatment reception significantly differed (*p* = 0.030), with black patients receiving treatment sooner than white patients.

Advanced stage NSCLC patients require appropriate timing of treatment [29] and personalized treatment regimens [35,36] to improve outcomes and enhance quality of life [35]. Personalized and timely care is still necessary, despite conflicting results. Given the constant evolvement of immunotherapy-based regimens, ongoing assessments are required to ensure evidence-based care is provided. As there is not an established consensus on an ideal TTI for treatment reception in stage IV lung cancer patients, future analyses are necessary to determine the optimal timing and its subsequent impact on patient outcomes.

There was no significant difference between black and white patients in the percentage of patients experiencing irAEs (*p* = 0.802). These findings are consistent with a study demonstrating a non-significant association between irAE occurrence and race [22]. There was no difference in platinum-based combination ICI therapy reception (*p* = 0.239, chi- square) or specific ICI selection (*p* = 0.363, chi-square) between black and white patients, indicating equitable treatment selection irrespective of race.

Although black patients have been shown to be diagnosed with lung cancer at a younger median age [37], our study did not establish any significant difference in median age at diagnosis (*p* = 0.600). However, black patients had a lower numerical median age of diagnosis compared to white patients (63.87 versus 65.62). When stratifying age at diagnosis (<65, 65–74, ≥75), there was a significant association between black and white patients and age at diagnosis (*p* = 0.036, chi-square), with a higher percentage of black patients being diagnosed at an earlier age. Consistent with prior studies [12,13], black patients in our cohort had fewer median pack-years (*p* = 0.025) at diagnosis than white patients. This has become an important metric as current screening guidelines may be considered too conservative, and may require alteration to identify more black patients at earlier stages [13].

Hospice enrollment is an essential component of patient care, especially in those diagnosed with advanced stage cancer. Hospice care has been shown to enhance quality of life, improve psychological symptoms, and decrease hospitalizations [38]. Black patients with advanced cancer have been shown to be enrolled in hospice care less often than their white counterparts, even when controlling for various factors such as age, sex, median household income, marital status, and site of primary cancer [39]. A systematic review assessing barriers to hospice care utilization in cancer patients demonstrated that racial minorities are less likely to utilize palliative or hospice care [40]. Conversely, our study found no difference in hospice enrollment between black and white patients (*p* = 0.279, chi-square).

Although race was expected to permeate patient outcomes, OS and PFS did not differ between black and white patients. Black patients received immunotherapy treatment sooner than white patients, highlighting that timely care was provided to this patient population. Awareness among health care professionals at our institution of barriers that black patients encounter may have helped narrow the gap. Future studies are warranted to elucidate the significance of these findings, and to better understand how race impacts TTI with an integrated analysis of other potential confounders. Equitable representation of diverse racial groups will be pivotal in understanding how race impacts cancer outcomes.

We acknowledge the limitations of our study such as its retrospective nature. Larger sample sizes are needed to better understand the non-statistically significant differences. These non-significant differences in survival may be due to insufficient powering of our study. Another potential limitation is that samples in this paper are from a single institution, which may have affected the results. Future research endeavors at other centers are warranted, especially those in other regions. Multi-institutional studies will offer more generalizable results. We recommend analysis of other parameters not captured in this retrospective review, such as the response rate (RR), to determine if race impacts results in this clinical context. However, we believe that our study has the following strengths: it exhibits near equal distribution of black and white patients, includes an examination of many demographic and clinical characteristics, and represents a patient population in which a majority received a regimen that is now the standard of care. This study highlights that in an equitable access healthcare system, race does not significantly impact the outcome of advanced lung cancer.

## 5. Conclusions

There was no impact of race on PFS or OS for patients receiving ICI-based therapy at our institution. There was no difference in median time to overall treatment initiation. The shorter time to immunotherapy treatment for black patients is an unexpected finding. Future prospective studies involving larger populations are required to validate current study results.

## Data Availability

Data are available in anonymized form upon reasonable request.
